# Partial Recurrent Laryngeal Nerve Paralysis or Paresis? In Search for the Accurate Diagnosis

**DOI:** 10.1155/2015/351704

**Published:** 2015-07-06

**Authors:** Alexander Delides, Panagiotis Kokotis, Pavlos Maragoudakis

**Affiliations:** ^1^Second Department of Otolaryngology, University of Athens, School of Medicine, “Attikon” University Hospital, 12462 Athens, Greece; ^2^Second Department of Neurology, University of Athens, School of Medicine, “Attikon” University Hospital, 12462 Athens, Greece; ^3^First Department of Neurology, University of Athens, School of Medicine, “Eginition” University Hospital, 11528 Athens, Greece

## Abstract

“Partial paralysis” of the larynx is a term often used to describe a hypomobile vocal fold as is the term “paresis.” We present a case of a dysphonic patient with a mobility disorder of the vocal fold, for whom idiopathic “partial paralysis” was the diagnosis made after laryngeal electromyography, and discuss a proposition for a different implementation of the term.

## 1. Introduction

The most commonly used terms to describe vocal fold mobility disorders are “paralysis,” meaning complete immobility, “paresis” for hypomobility, and “fixation” that describes an immobile vocal fold due to mechanical obstruction of its movement, such as arthritis of the cricoarytenoid joint or scarring of the posterior commissure.

“Paralysis” and “paresis” are still widely used terms even though diagnosis for the neurogenic origin of the disorder is not always confirmed by laryngeal electromyography (LEMG) [1].

Neurogenic compromise of vocal fold function exists along a continuum encompassing vocal cord hypomobility (paresis) to vocal fold immobility (paralysis) with varying degrees and patterns of reinnervation. Vocal fold paralysis (VFP) may result from injury to the vagus or the recurrent laryngeal nerves anywhere along their course from the brainstem to the larynx. Phenomena of synkinesis are encountered in the human larynx leading to movement disorders in a reinnervated vocal fold after injury to the recurrent laryngeal nerve, the nerve of both abduction and adduction.

The most common causes of mobility disorders are iatrogenic, after surgery in the structures that lie along the course of the inferior laryngeal nerves. The second most common cause is “idiopathic,” meaning an unknown aetiology.

We hereby present a case where a partial recurrent laryngeal nerve paralysis was confirmed by laryngeal needle electromyography (LEMG).

## 2. Case Report

The patient, a forty-five-year-old otherwise healthy male, was referred to the Voice and Swallowing Clinic of the “Attikon” University Hospital for a sudden onset of dysphonia that had occurred fifteen days priorly. A mobility disorder of his left vocal fold had been diagnosed and had already undergone a complete radiologic workout including a brain and cervical MRI as well as a CT of the thorax and Ultrasound of the thyroid, all of which were normal. His past and recent medical history was free of viral infections, trauma, or any other medical condition.

**Figure 1:**
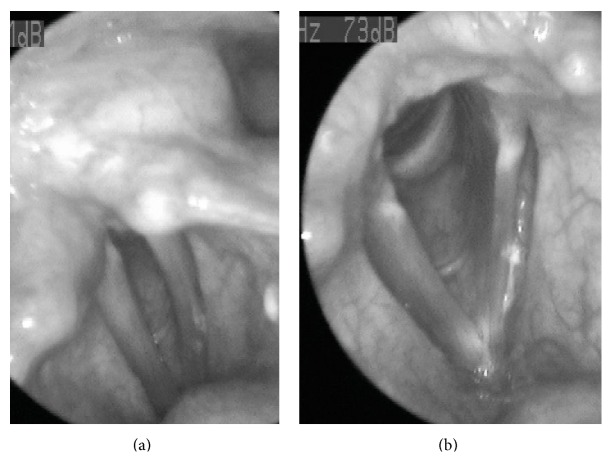
Laryngoscopic images during phonation (a) and inspiration (b).

A complete ENT examination was performed prior to laryngeal endoscopy that was free of any pathological findings. Upon rigid endoscopy and videostroboscopy, his right vocal fold appeared normal with normal range of motion. On the left side, the body of the true vocal fold was immobile and fixed to a lateral position whereas the arytenoid seemed to be having a full range of motion (Figure 1). His voice was breathy due to the glottal incompetence and was unable to sustain a maximum phonation time (MPT) more than 2.4 seconds.

An initial diagnosis of vocal fold myopathy was proposed and the patient was referred for a complete neuromuscular workout that included repetitive nerve stimulation test for myasthenia gravis and anti-acetylcholine serum antibodies. All were normal.

**Figure 2:**
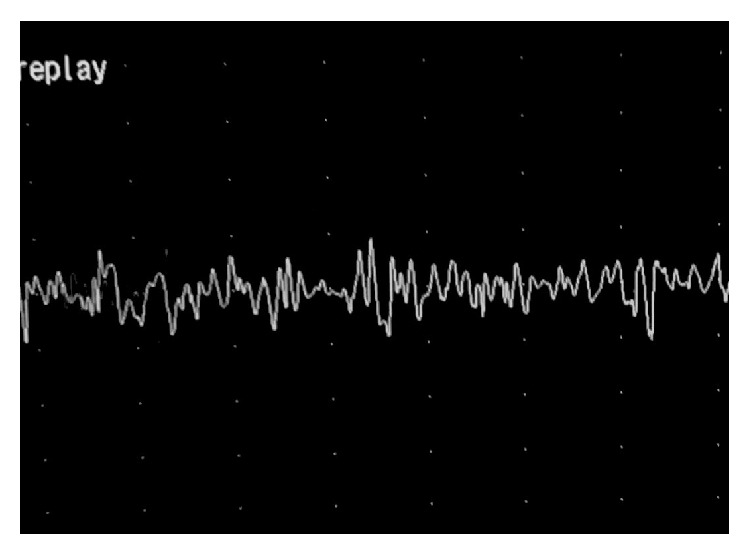
Laryngeal electromyography of the normal LCA.

**Figure 3:**
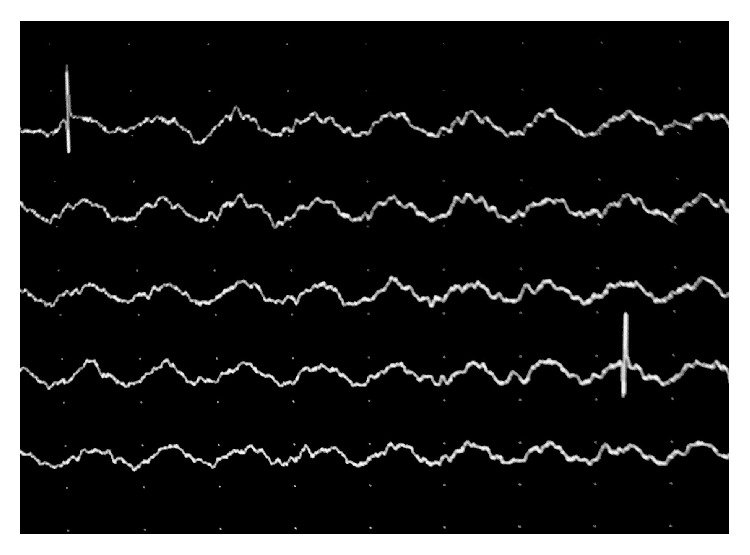
LEMG of the denervated TA muscle.

Laryngeal electromyography was performed with the use of bipolar needle electrodes that included bilateral testing of the cricothyroid (CT) muscles, the thyroarytenoids (TA), and lateral cricoarytenoids (LCA). All right side (normal) muscles did not reveal any kind of denervation potentials and recruitment patterns, upon voicing with sustained “i” and “e,” were within normal range for these muscles, including motor units morphology. Normal motor units potentials and recruitment pattern were also produced in the CT and LCA on the affected side (Figure 2), whereas a pattern of denervation indicated by sharp fibrillation and positive potentials were shown in the TA muscle (Figure 3). The motor units were of small amplitude and their recruitment pattern significantly reduced. These findings were only consistent with a diagnosis of “partial inferior laryngeal nerve paralysis” that involved only the branch innervating the thyroarytenoid muscle.

The patient was followed for a total period of nine months, after which endoscopic examination revealed a similar movement pattern of the larynx. His glottal gap had improved, as had his breathiness. Nevertheless his MPT was still 3.6 seconds and was still experiencing symptoms of severe dysphonia progressing during the day as well as vocal fatigue. He underwent an injection laryngoplasty procedure with hydroxylapatite (Radiesse) under local anesthesia in the office with the transcutaneous hyothyroid approach, after which his MPT was raised to 29 seconds. Three months later, a follow-up telephone call was made, confirming his voice to be close to normal. He was pleased with his voice and did not experience any vocal fatigue symptoms.

## 3. Discussion

“Partial recurrent laryngeal nerve paralysis” is a diagnosis inconsistent with the spectrum of vocal fold immobility disorders that have been proposed to date. Partial paralysis for most authors is identical to paresis, meaning that all muscles innervated by the ILN are equally or almost affected. Most authors agree to the use of the term “vocal fold motion impairment” [2, 3].

The vocal folds are vaguely characterized by most as paretic, paralytic, and fixed, with the former two types involving the vast majority of diagnoses, since LEMG is a prerequisite to confirm the later, a tactic uncommon in everyday practice. “Paresis” to most physicians' minds is equal to “hypofunction” or less movement compared to the other side. Such an interpretation means that all or most of the muscles innervated by the RLN are somewhat equally affected by the causing factor that leads to the dysfunction.

In our discipline, other cranial nerve functional disorders such as those involving the 8th or 7th nerve are frequently encountered with a partial dysfunction of them that leaves some areas of innervation completely normal, whereas others are dysfunctional. Hearing loss is seldom manifested with a uniform loss of all frequencies and peripheral facial nerve paresis (or paralysis) of traumatic or viral origin is often diagnosed with a partial loss of movement of some of the muscles innervated by it [4].

We do know however that such a simplification is not justified by the anatomy and physiology of the inferior laryngeal nerve, consisting of fibers innervating different adductors and abductors. In the physical history of reinnervation, synkinesis occurs, as does it in other cranial nerves such as the facial nerve. In the larynx, synkinesis might extremely lead to adduction in inspiration and abduction in phonation but usually causes persistent dysmotility due to simultaneous stimulation of abductor and adductor muscle fibers [1, 5].

A “partial paralysis” would more accurately imply that some of the nerve fibers have completely lost their functionality, possibly ending in a complete loss of function (paralysis) of some of the muscles but a sustained function of others.

It is thus possible to make the argument that a partial loss of nerve fibers within the trunk of the nerve, as is encountered with the reverse regeneration process in cases of synkinesis, might be manifested as a partial loss of function of some of the muscles involved in vocal fold motion [4].

These assumptions are poorly supported by the literature and our case is by no means a proof of an existence of such a pathological condition. But we fail to conclude that our case may fit in any of the known categories of vocal fold immobility disorders and we believe that more research is needed to lighten our knowledge of neurophysiology of the larynx.

## References

[1] Blitzer A., Jahn A. F., Keidar A. (1996). Semon's law revisited: an electromyographic analysis of laryngeal synkinesis. *Annals of Otology, Rhinology and Laryngology*.

[2] Crumley R. L. (2000). Laryngeal synkinesis revisited. *Annals of Otology, Rhinology & Laryngology*.

[3] Benjamin B. (2003). Vocal cord paralysis, synkinesis and vocal fold motion impairment. *ANZ Journal of Surgery*.

[4] Sulica L. (2008). The natural history of idiopathic unilateral vocal fold paralysis: evidence and problems. *The Laryngoscope*.

[5] Maronian N. C., Waugh P., Robinson L., Hillel A. D. (2004). A new electromyographic definition of laryngeal synkinesis. *Annals of Otology, Rhinology and Laryngology*.

